# Phylogenetic placement of metagenomic reads using the minimum evolution principle

**DOI:** 10.1186/1471-2164-16-S1-S13

**Published:** 2015-01-15

**Authors:** Alan Filipski, Koichiro Tamura, Paul Billing-Ross, Oscar Murillo, Sudhir Kumar

**Affiliations:** 1 Institute for Genomics and Evolutionary Medicine, Temple University, Philadelphia, PA 19122, USA; 2 Department of Biological Sciences, Tokyo Metropolitan University, Tokyo, Japan; 3 Department of Molecular Biology and Genetics, College of Liberal Arts and Sciences, Cornell University, Ithaca, NY, 14853-5905, USA; 4 Department of Biology, Temple University, Philadelphia, PA 19122, USA; 5 Center for Genomic Medicine and Research, King Abdulaziz University, Jeddah, Saudi Arabia

## Abstract

**Background:**

A central problem of computational metagenomics is determining the correct placement into an existing phylogenetic tree of individual reads (nucleotide sequences of varying lengths, ranging from hundreds to thousands of bases) obtained using next-generation sequencing of DNA samples from a mixture of known and unknown species. Correct placement allows us to easily identify or classify the sequences in the sample as to taxonomic position or function.

**Results:**

Here we propose a novel method (*PhyClass*), based on the Minimum Evolution (ME) phylogenetic inference criterion, for determining the appropriate phylogenetic position of each read. Without using heuristics, the new approach efficiently finds the optimal placement of the unknown read in a reference phylogenetic tree given a sequence alignment for the taxa in the tree. In short, the total resulting branch length for the tree is computed for every possible placement of the unknown read and the placement that gives the smallest value for this total is the best (optimal) choice. By taking advantage of computational efficiencies and mathematical formulations, we are able to find the true optimal ME placement for each read in the phylogenetic tree. Using computer simulations, we assessed the accuracy of the new approach for different read lengths over a variety of data sets and phylogenetic trees. We found the accuracy of the new method to be good and comparable to existing Maximum Likelihood (ML) approaches.

**Conclusions:**

In particular, we found that the consensus assignments based on ME and ML approaches are more correct than either method individually. This is true even when the statistical support for read assignments was low, which is inevitable given that individual reads are often short and come from only one gene.

## Background

Rapid and inexpensive sequencing methods yielding short reads have become common for analyzing mixed-species biological samples [1-8]. Phylogenetic and taxonomic classification of species present may be done by extracting and amplifying fragments of a distinctive gene such as one for ribosomal RNA from the sample and comparing the results to reference samples [9,10]. Early methods for identification of metagenomic reads were based on BLAST [11,12], but these approaches do not define the best phylogenetic placement [e.g., [13,14]]. Consequently, rigorous phylogenetic methods under the maximum likelihood (ML) principle have been developed for ascertaining the phylogenetic placement of a sequence read in a given species tree [15-17]. However, an approach using the Minimum Evolution (ME) principle is not yet available for classifying metagenomic reads, which is important because, as we show below, it is possible to develop a method that does not require heuristics for classifying reads when using a matrix of pairwise distances. Furthermore, methods employing different optimality principles (ML and ME) can be useful in molecular phylogenetics to assess the robustness of inferences to underlying biases of individual methods.

Therefore, we have developed a distance-based approach (called *PhyClass*) under the Minimum Evolution (ME) principle for classifying metagenomics reads. We have also implemented an efficient procedure for producing bootstrap statistical support for the assignment of any read to any position in the reference species tree. In the following, we first describe the new method and then assess the absolute accuracy of the new approach by using computer simulations. We also compare the performance of *PhyClass* with the most accurate existing Maximum Likelihood based placement program, EPA [15]. EPA is based on the RAxML package [18] and has been shown to perform as well as or better than other methods for this purpose [15,16]. We also discuss the usefulness of concordance between *PhyClass* and EPA inferences in identifying correct assignments even when the statistical support for the read assignment is low.

## Results and discussion

### Details of the Algorithm

In the *PhyClass* method, the input consists of a set of partial sequences of a specific gene/genomic segment (reads), a reference tree topology (T) describing the phylogenetic relationships among some set of *n* species, and a multiple sequence alignment (MSA) of the relevant genes or genomic segments for these *n* species. For a given read *r*, the goal is to find a best-fit placement for *r* in the tree T. This may be done by minimizing the cost, defined as the sum of branch lengths *S_b_*_,_*_r_* of the tree containing read *r* attached to branch b of T, over all possible placements *b*. Under the ME principle, the configuration where *S_b_*_,_*_r_* is the smallest is the best placement [19]. In this calculation, we use a matrix *D* of distances among all *n*+1 sequences (reference alignment plus the given read). For all placements of a read, it is only necessary to recalculate pairwise distances between the given read and the *n* reference sequences, with the pairwise distances among the reference sequences calculated once at the start of the analysis. Furthermore, the calculation of *S_b_*_,_*_r_* for all placements of the same read in a fixed reference tree can be done efficiently, because calculating the change between *S_b_*_,_*_r_* and *S_b’_*_,_*_r_* where *b* and *b’* are adjacent branches of T, requires only a limited calculation involving local branch lengths [19,20]. Therefore, no approximate heuristics are necessary to efficiently apply the ME principle by evaluating all possible topological locations for read *r*. Different distance measures may be used for the ME computations.

### Accuracy of the Method Using Complete Sequences

**Figure 1:**
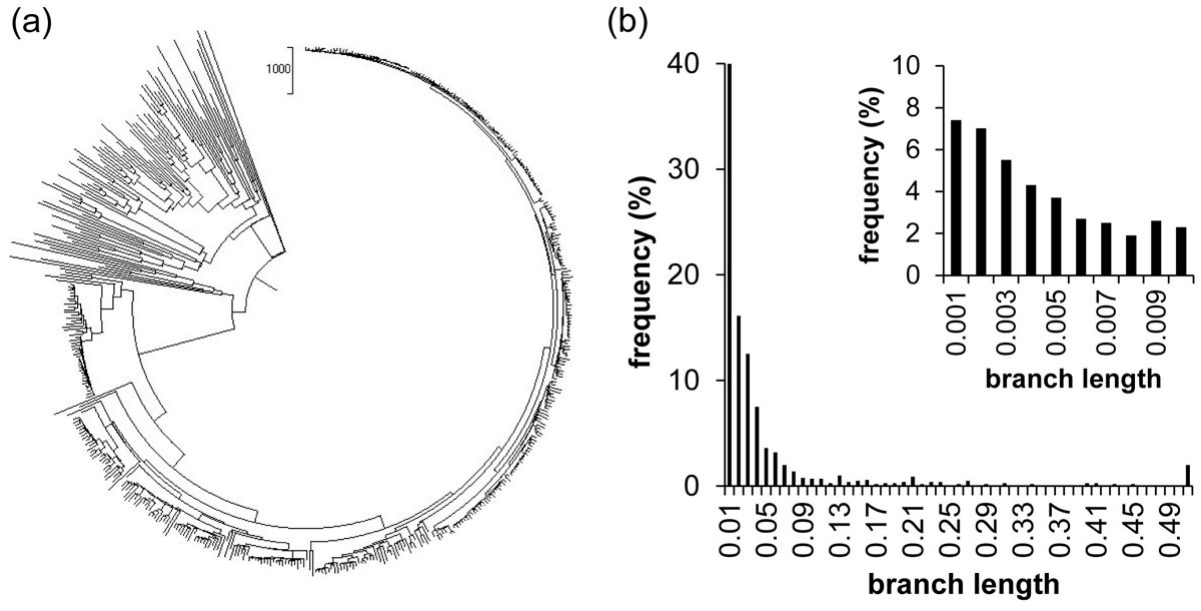
**Primary data set.** 1a) depicts the model tree for generating the sequences for the S500R data set. 1b) shows a histogram of branch lengths (substitutions per site) of the S500R tree. The detail shows the finer division of the first bin of the larger graph. 7.4% of branches, for example, have length less than or equal to 0.001 substitutions per site.

We evaluated the performance of the *PhyClass* approach using simulated datasets containing 500 sequences (each sequence 2000 base pairs long). The dataset is modelled after observed rRNA evolutionary parameters, with evolutionary rate varying extensively among lineages in the model tree (Figure 1) resulting in a tree containing a few long branches and many short ones. This tree was produced by starting with an ultrametric tree of 500 arbitrarily selected taxa and then independently varying each branch’s rate over a uniform distribution from 0.11 substitutions per billion years (Gy) to 0.33 substitutions/Gy (plus or minus 50% of the estimated mean nominal rate of 0.22 substitutions/Gy). We used Seq-Gen to produce sequence data sets from this tree [21]. Results described below are based on mean ± one standard deviation of results from ten data sets.

We began by establishing a baseline profile, where we assumed that the original full length sequence was available (2000 base pairs) along with the true MSA as query. In this way, we established the maximum possible accuracy one could achieve if the metagenomics sequence extraction process produced complete full-length error-free sequences that could be aligned perfectly. We used each individual sequence in the reference tree of 500 sequences as a read (query) for metagenomic analysis by first removing that sequence from the tree and then evaluating the ability of *PhyClass* to replace it at the correct topological position in the tree of 499 remaining taxa. Under these conditions, 66% (± 1.7%) of the original perfect sequences could be re-assigned correctly (*f*_c_ = 66%).

**Figure 2:**
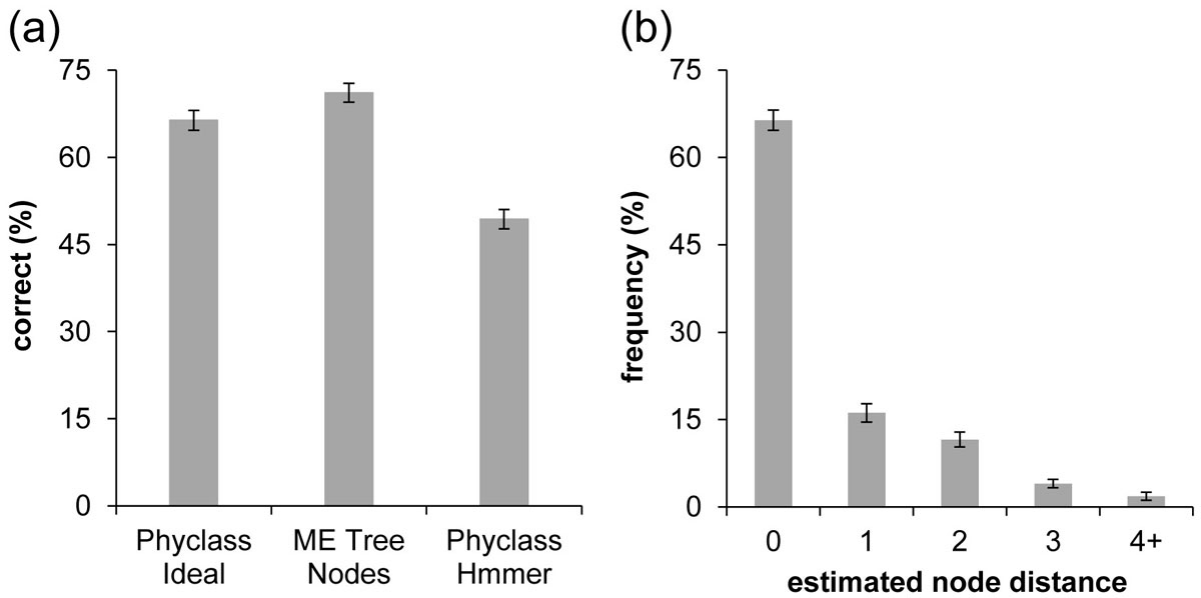
**Accuracy of new methods for the full-length case.** 2a) In the ideal case (full-length queries with true alignment and no artificially introduced noise) *Phyclass* classified 66.4% of them correctly, with a standard deviation of 1.7%. For comparison, we show (second bar) the number of taxa placed correctly under Minimum Evolution phylogenetic analysis of the data set (71.1% correct, with a standard deviation of 1.6%). The third bar shows the percentage correct (52.2%, with standard deviation of 1.4%) when *Phyclass* was run on the same sequence set aligned by HMMER. 2b) Shows the distribution of node distances for classifications in the ideal case (Node Distance is defined as the number of nodes in the phylogenetic tree intervening between the correct placement and the inferred placement.) We see that 47% of incorrect estimates are off by only one node in the tree . Error bars represent ± 1 standard deviation of individual estimates, based on ten replicates.

While this value of *f*_c_ may appear low given that the data is ideal, it is not surprising because single genes with limited sequence length are known to yield phylogenetic results with rather limited accuracy [22-24]. This will particularly be the case for short branches because of the effect of sampling error on the ability to bound the length estimates away from zero, which is often the case when highly conserved sequences are used. In fact, the proportion of branches correct in inferred ME trees obtained using the complete and perfect datasets was similar (71% vs. 66%) to the *PhyClass* results (Figure 2a), so the *Phyclass* placement error rate is reasonable.

### Effect of Alignment

As the true alignments and perfect sequences are never available in a real metagenomics context, we next examined the performance when using HMMER for aligning query reads to sequences in the reference MSA [25]. For added realism, we inflicted three types of errors to the query sequences: 1% of the bases were mutated to another base, another 1% bases were deleted, and 1% bases were duplicated (stutter) [26,27]. The resulting accuracy of 49.4% was lower than the ideal case (Figure 2a) (*f_c_* = 49.4 ± 1.6%).

It is important to realize that the true estimate of the metagenomics accuracy will generally be higher than the *f*_c_ we report here (for a given sequence length), because, in our evaluations species corresponding to our queries were never allowed to appear in the reference tree. That is, we removed the query sequence from the reference tree and alignment before using *PhyClass*. Otherwise, we expect to be able to correctly assign the query to its source data due to the small evolutionary distance between the query and the correct reference sequences. This was confirmed by our analyses, where the *f_c_* for known sequences (2000 bp queries with simulated read error and HMMER alignment) was 97.2% for the simulated dataset. Therefore, the metagenomics read assignment accuracy will be considerably higher when the proportion of sequences from known species is high. Nevertheless, in order to make the tests as challenging as possible, we introduced read errors as described above, applied HMMER instead of using true alignment information, and conducted analyses after deleting the true target species from the tree in all subsequent analyses reported below. In this sense, our results are worst-case scenarios. A similar protocol was used in the study by Berger et al. [15].

### Anatomy of Misplacements

In order to better understand the anatomy of misplacements, we analyzed the proximity of the erroneous assignments to correct location by recording the number of intervening nodes separating the correct branch and the branch assigned by *PhyClass* (Node separation, *S*_N_) and the evolutionary distance, in terms of mean number of substitutions per site, spanned by the correct and incorrect placement (Branch length separation, *S*_B_). For the correct placements, both of them were 0. We found that 43% of the incorrectly placed reads were assigned to a branch immediately adjacent to the correct branch (*S*_N_ = 1) and the other 40% were assigned on branches that were just two nodes away (*S*_N_ = 2; Figure 2b). Thus, approximately 94% of these placements were made no more than two nodes from their correct position in the tree.

In ordinary (non-testing) use, evolutionary distance information can also be useful. For example, if the unknown read is placed into the tree at a long distance from the nearest reference node, then we know that the item has been placed into a relatively isolated phylogenetic position with respect to the reference samples. This information, which is readily available as all branch lengths in the tree are automatically computed, may be useful as an indicator of reads from anomalous species.

### Analysis of Partial Sequences

**Figure 3:**
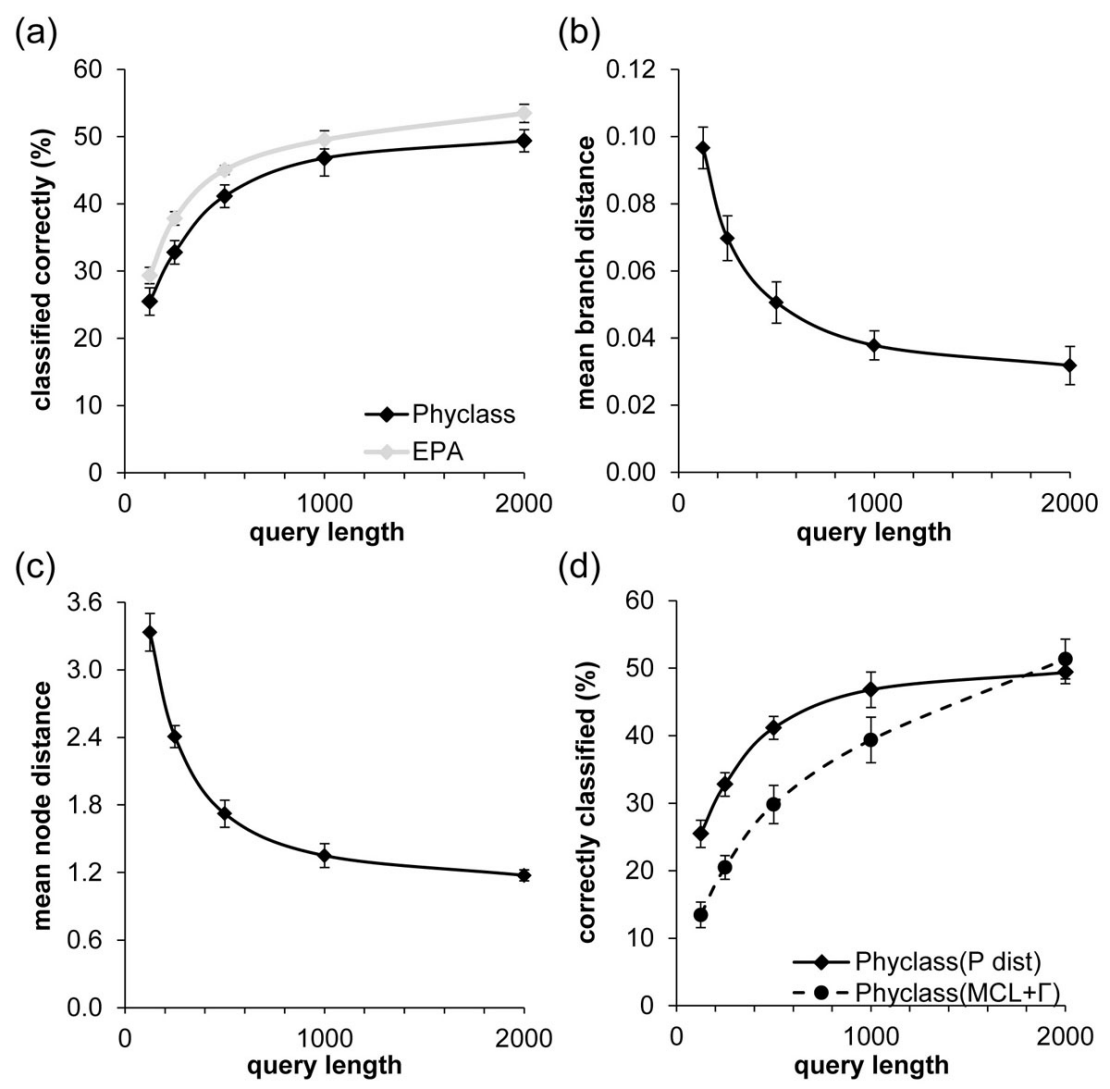
**Accuracy of new methods for varying sequence lengths.** 3a) shows percentage of queries classified correctly by query length. The black line represents *Phyclass* and the gray line represents EPA. EPA accuracy exceeds that of *Phyclass* by an average of 3.9 percentage points correct for all query lengths tested. 3b) and 3c) show the decrease in mean branch distance (evolutionary distance between true placement and inferred placement) and mean node distance (number of tree nodes between true placement and inferred placement) , respectively, as query length increases. 3d) shows a comparison of accuracy rate between ordinary *Phyclass* (which uses p-distance) and *Phyclass* using a sophisticated distance measure (MCL + Γ). Except for the longest queries, P-distance provides better average classification rates.

The analysis of full length sequences as described above represents an ideal case. In actual metagenomics samples, however, only partial sequences are recovered that may be as short as 100 base pairs. Therefore, we examined the accuracy of *PhyClass* when only partial sequences were available. In this case, reads of different sequence lengths (125, 250, 500, 1000, and 2000) were extracted from the full length sequences by randomly sampling contiguous blocks of sites. For each of the 500 species, one sample of each length was extracted, the different kinds of noise were applied, and the resulting simulated query was aligned to the full reference set. As expected, the shortest reads show the lowest assignment accuracy (Figure 3a). The accuracy trends showed a log-linear relationship with read length. With a 16-fold decrease in read length (from 2000 to 125), the fraction of correct assignments is halved. Similar trends, but in the inverse direction, were seen for the branch length separation (Figure 3b) as well as node separation per incorrect assignment (Figure 3c).

### Distance Measures

We hypothesized that an additional factor affecting accuracy is evolutionary distance measure used, although it was not clear *a priori* what the effect would be, as more sophisticated evolutionary models reduce distance estimation bias, but also increase the variance of the estimate [28]. For the above results, we used *p*-distance (fraction of sites different between two sequences), which has a relatively small estimation variance and is computable for all sequence pairs, unlike more sophisticated distance measures that often fail (e.g. Jukes-Cantor distance for sequence pairs that differ at more than 75% of sites [29]). It is known that *p*-distance is a good approximation to more sophisticated model-based distances over short evolutionary distances, and it is generally these distances we are most concerned with when making difficult differentiations among closely-placed nodes on short branches [30]. Use of *p*-distance in an ME context is further supported in several studies which found that, unless sequence lengths are very great, the simple *p*-distance generally gives better results in phylogenetic inference than more complicated distance measures [31,32]. In agreement with these results, empirical tests using *PhyClass* on our data sets show that *p*-distance performed slightly better than more sophisticated distances in terms of placement accuracy. For shorter sequences, *p*-distance provided improved accuracy over a very sophisticated distance measure (Figure 3d) based on the Maximum Composite Likelihood method [33,34] with a gamma model to account for rate variation among sites [35], which matched the Tamura-Nei model [36] used to generate the sequence data according to the tree.

### Significance of placements

We assess the statistical significance of the placement using the bootstrap resampling procedure, where the *n* pairwise distances between the given read and the *n* reference sequences in the MSA are obtained by using multinomial sampled counts of 16 (4×4) possible nucleotide pairs for each read-reference sequence comparison. Therefore, the pairwise distances in *D* corresponding to the read-reference pairs are updated in bootstrap replicate based on the multinomial counts, with rest of the pairwise distances, ½ *n*×(*n*-1), between reference sequence pairs remaining the same in every bootstrap replicate, because the topology of the reference tree is assumed to be fixed in each replicate. We used *p*-distances, as bootstrapping for these distances is fast to compute using the multinomial sampling.

**Figure 4:**
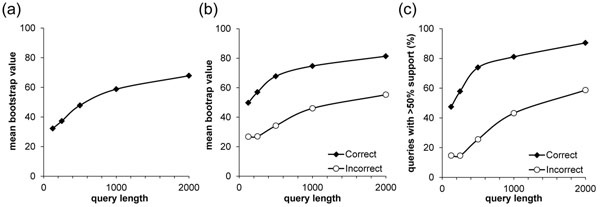
**Results from the bootstrap analyses.** 4a) Average bootstrap value by query length over all assignments, correct or incorrect. 4b) Average support for right and wrong assignments 4c) Percentage of correct and incorrect assignments receiving at least 50% support.

As expected, higher average bootstrap support was seen for the longer reads (Figure 4a). More importantly, the fraction of correct placements receiving high bootstrap score was much higher than the wrong assignments (Figure 4b). But, still, there were many misclassifications receiving support of at least 50% (Figure 4c). A reason for correct placements without high bootstrap support and incorrect placements with more than 50% bootstrap support was the paucity of substitutions in the section of the reference alignment that overlaps with the query sequence. This problem of zero or effectively zero branch lengths cannot be remedied by any method, because there is no information in the data for correctly placing queries when they come from such branches [28]. When the correct placement of a query requires insertion into such a branch, attempts at placement only contribute to noise, which obscures efforts to compare results under different conditions. Therefore, the bootstrap support values, and those obtained using other confidence assessment approaches, need to be used carefully when the placement of a read is at or adjacent to a very short branch.

### Additional Data Sets

**Figure 5:**
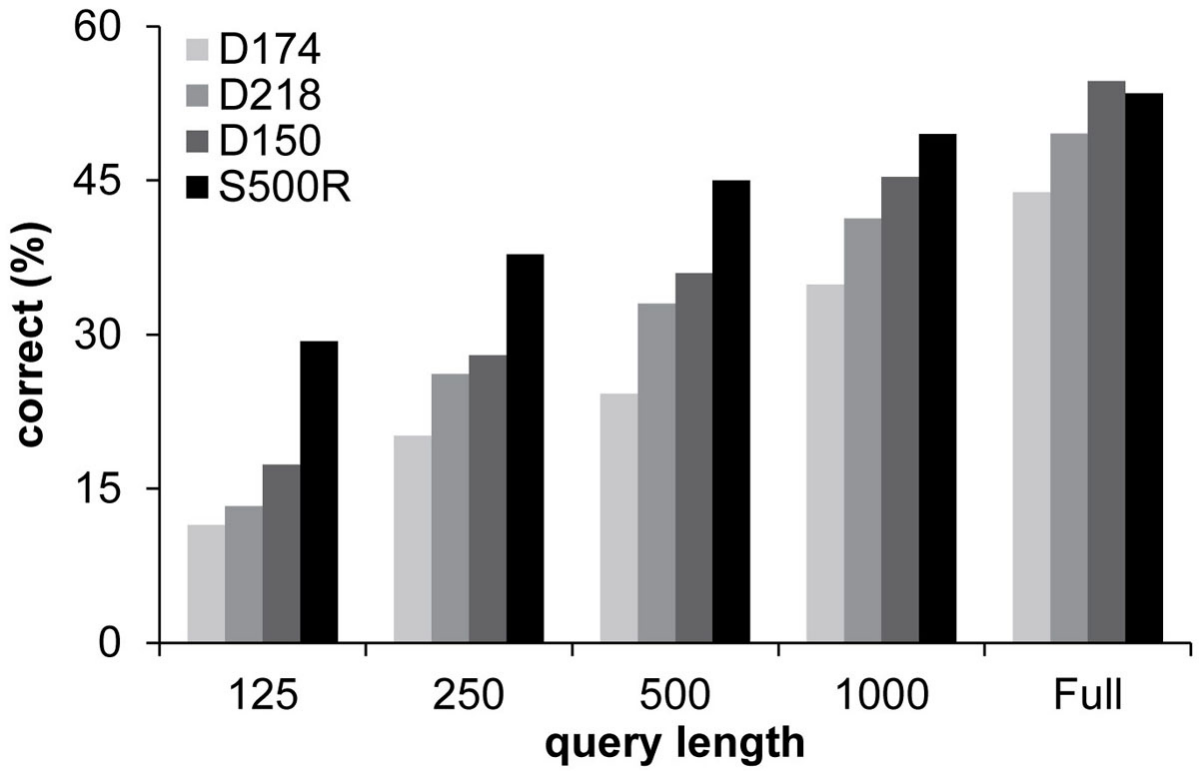
**Accuracy for more data sets.** This figure compares accuracy for all test data sets, by query length. We see a consistent trend toward lower accuracy with shorter queries, but also a substantial difference in accuracy among sets that persists at all query lengths. Set D218 is consistently worst, perhaps due to the fact that it has a much larger proportion of indels (35%) than the other sets (at most 6%).

We performed testing using four additional data sets. Three of these were taken from a similar study in the literature [15]. These are based on empirical sequence data sets. These sets were aligned and a ML tree for them constructed as described in that paper. In the absence of knowledge of the true phylogeny of the sequences, these trees were taken as truth for the purpose of determining accuracy. The fourth data set was simulated using the same tree topology as in the primary example but with constant evolutionary rate (see Materials and Methods). Figure 5 shows overall percentage correct for all sets by query length. Note that there is considerable variation among the different sets (most pronounced for shorter query lengths), with the set that we call D218 the most difficult to classify. This may be due to the many indels in the sequences for that set (see Table 1)—only 688 of the 2294 sites have complete coverage.

**Table 1:** Characteristics of test data sets.

Set	Sequence length	Ref. sequence	Mean branch length	Indels (%)	Diameter	Origin
D174	1241	714	0.03	0.06	2	Empirical
D218	2294	218	0.12	35	3.3	Empirical
D150	1269	150	0.06	5	3.4	Empirical
S500R	2000	500	0.05	0	2.3	Simulated

Note.- Sequence length is the length of the original alignment in nucleotide sites (including indels). Ref. sequences refers to the number of sequences in the alignment. Mean Branch Length is the average length (in substitutions per site) of branches in the original tree. Indels is the percentage of sites in the alignment that are hyphens or question marks or IUPAC *N* metacharacters, representing any nucleotide. Tree Diameter is the maximum patristic evolutionary distance (total substitutions per site along the branches of the tree) between any two taxa in the tree. Source refers to the origin of the sequences.

### Comparison with EPA

In order to assess the value of *Phyclass* in the context of the state of the art, we compared its accuracy with EPA which is widely used. EPA chooses optimal placement using a heuristic approximation of the total Maximum Likelihood (ML) of the candidate trees [15]. It is based on the widely-used ML tree inference program RAxML [18]. The accuracy of EPA was consistently approximately 4 error percentage points higher than *PhyClass* over all query lengths for the model S500R data set and a similar amount on the empirical data sets (Figure 3a). This is consistent with slightly better performance of likelihood-based methods over ME methods (e.g. [36]).

### Speed

Although our goal here is to evaluate the accuracy of the *Phyclass* method, and not to do time optimizations, especially if they involve heuristic shortcuts that may lead to sub-optimal placement, we made some observations about timings. Currently, our prototype *Phyclass* classifier, exclusive of one-time initial alignment using HMMER, takes around 6 seconds per query to compute one placement on the S500R data set. This is compared to the performance of EPA with the “slow” option (which we used for all accuracy tests because it provides the highest accuracy) which used around 14 seconds per query. (EPA also provides a suboptimal “fast” option, which uses about 0.3 seconds/query after an initial setup time of 4 minutes or so for the entire set of queries.) There are several clear opportunities for time optimizations in *PhyClass* without compromising the current strictly optimal ME calculation. In our prototype version of *PhyClass*, each query is processed completely independently and most time is spent in reading and writing text files, setting up data structures and formatting them for output. This is appropriate, since, because of the test mode requirement of removing the true source taxon from the tree and the reference set to see if it is re-inserted at the same point, the data changes with each query. Most of this can be eliminated in more typical use by running a large number of queries in batch mode so that most computation (e.g. pairwise distances among reference sequences) can be factored out of the per-query workload into a setup phase and held in computer memory rather than written to external files. An analysis of the 6-second runtime of *PhyClass* on the S500R data set shows that more than half is consumed reading and writing text files, with the remainder divided between distance and sum-of-branch-length calculations, the former of which can be factored to a set-up phase instead of being done on a per-query basis. EPA already incorporates optimizations to pre-compute and retain in memory as much as possible.

### Phyclass and EPA used together

**Figure 6:**
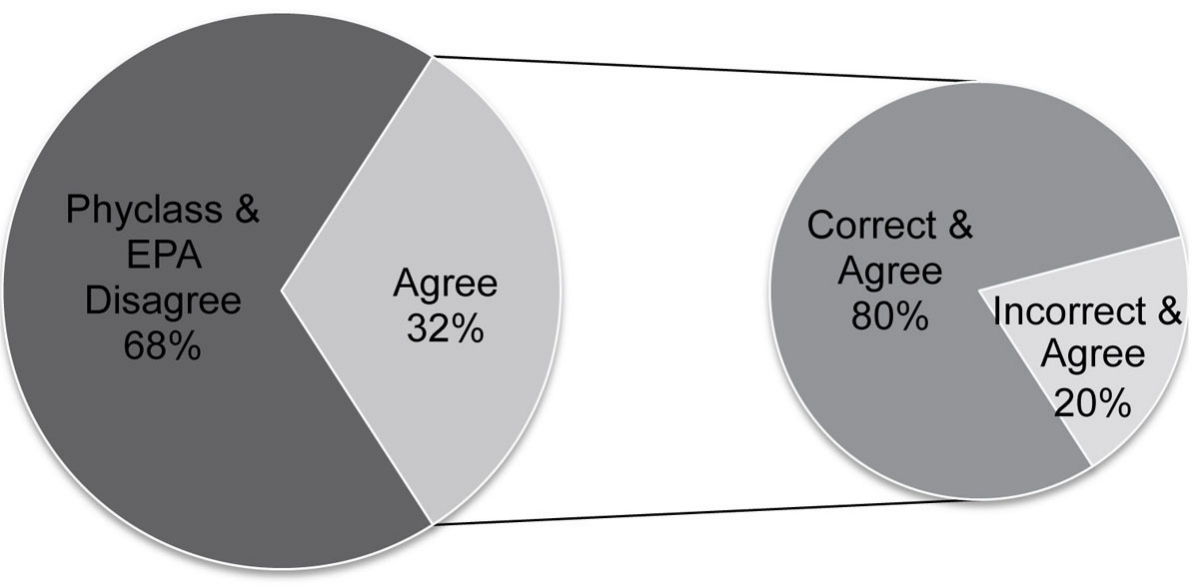
**Joint application of ME and ML methods.** We can obtain 80% correct classification on 32% of the data by using both methods together. Based on a mixture set of queries of all lengths from the S500R data set, EPA got 41% correct and *Phyclass* got 40% correct. They were both in agreement, correctly or incorrectly, on 32% of queries. Among the agreeing queries, the ratio of correct to incorrect queries is 4 to 1, that is, among these 32%, 80% are correct. In this way, by running the two classification methods in parallel, it is possible to roughly double the accuracy for about a third of the queries by running both methods in parallel.

Finally, we explored whether placement can be improved by using both methods discussed above (*Phyclass* and EPA) in conjunction with each other (Figure 6). We found that, using a mixture of equal numbers of query lengths (125, 250, 500, 1000, 2000) based on the S500R data set, *Phyclass* alone correctly classified 40% of queries, and EPA correctly classified 41%. However, for the 32% of the data for which *Phyclass* and EPA agreed, correctly or incorrectly, on the placement, 80% is classified correctly. In this way, we are able to roughly double the accuracy rate for about a third of the queries. This dual approach may be considered an advantage over traditional bootstrapping, which is usually conservative and time consuming.

## Conclusions

Using sequence simulation, we evaluated the use of the Minimum Evolution principle to place aligned fragmented DNA sequences representing metagenomic reads into phylogenetically correct positions in an existing tree of known sequences. Our tests showed the accuracy of the new method to be comparable to, but in most cases slightly less than that of the best existing Maximum Likelihood program to deal with the same problem. Consensus assignments based on both approaches together were found to be often more correct than either method individually, even when the statistical support for assignments was low.

## Methods

We used four primary data sets for testing, three empirical and one simulated. The empirical ones (D150, D218, and D714) were from the EPA study [15]. Inferred ML trees and reference sequences were downloaded from the site associated with that report.

We simulated data set S500R by sampling 500 terminal taxa, including bacteria, archaea, and eukaryota from a 1610-taxon tree that represented all major groups from the tree of life [37]. 2000-bp DNA alignments for the tree were simulated, without indels, using parameters calculated from the Silva rRNA dataset [38] using Seq-Gen [21]. Using the model testing feature of MEGA [39], the best model for these sequences was calculated to be the GTR + Γ + I with an alpha value of 0.7, five categories for the gamma rate distribution, and 0.8% invariant sites. A nominal evolutionary rate of 0.22 substitutions/Gy was used to produce an alignment with similar pairwise distances to the Silva empirical data [38,40]. Variation was added to the rates by independently varying each branch’s rate in the tree over a uniform distribution from 0.11 substitutions/Gy to 0.33 substitutions/Gy (plus or minus 50% of the nominal value). Table 1 shows some empirical characteristics of these data sets.

The EPA program was obtained from the developers’ site. EPA provides a “slow” and “fast” placement option. According to the developers, the “slow” option is more accurate [15]. We confirmed this with a test sample and therefore used EPA “slow” mode for all accuracy comparisons.

All placement accuracy statistics for EPA and *Phyclass* were calculated by first deleting from the tree and the reference sequence alignment the reference sequence from which the query was extracted. Then the placement of that query is counted as correct if it is inserted into the same branch to which the original sequence had been attached. This is the same testing strategy as used in Berger et al. [15].

We found HMMER in profile alignment mode (http://hmmer.janelia.org/), to be faster and more accurate than other alignment programs, including Muscle [41] and CLUSTAL[42]. The EPA developers recommended HMMER as well. We conjectured that alignment method is an important factor in placement accuracy. We tried many different alignment methods while developing this method and found that HMMER profile alignment was the best performer in terms of both speed and accuracy of results. Since the testing method involved extracting queries from known reference sequences, we had available the true alignments for comparison. We found, unexpectedly, that the average difference in percentage of exact placement, over all data sets and categories, between HMMER profile alignment and true alignment is only 3.2 percentage points, suggesting that not much is to be gained, in terms of accuracy, at least, by alignment improvements.

The MEGA5 and MEGA-CC phylogenetics software packages were used for all phylogenetic analyses and distance computations [39,43].

## Competing interests

The author(s) declare that they have no competing interests.

## Authors' contributions

Primary conceptual work and direction was provided by S.K. and A.F., while K. T. and A.F. contributed to implementing the *PhyClass* algorithm. A.F., S.K, O. M., and P. B-R. were primarily responsible for simulation programming and data analysis.

## Declarations

Publication charges for this article have been funded from research grants from National Institutes of Health (NIH; HG002096-12) and HiCi-1434-117-1 from KAU.

This article has been published as part of *BMC Genomics* Volume 16 Supplement 1, 2015: Selected articles from the 2nd International Genomic Medical Conference (IGMC 2013): Genomics. The full contents of the supplement are available online at http://www.biomedcentral.com/bmcgenomics/supplements/16/S1
